# The Dual Burden of Hepatitis B and C Among Drug Users in Asia: The First Systematic Review and Meta-Analysis

**DOI:** 10.3390/pathogens14040360

**Published:** 2025-04-07

**Authors:** Ali A. Rabaan, Kizito E. Bello, Zaheda Radwan, Amal K. Hassouneh, Hayam A. Alrasheed, Jawaher Alotaibi, Bashayer Basrana, Ali A. Zaidan, Mohammed A. Garout, Tasneem I. Zaidan, Kawthar Amur Al Amri, Sana A. Alshaikh, Kawthar Haider Al Alawi, Razi A. Alalqam, Huseyin Tombuloglu, Nabiha A. Bouafia

**Affiliations:** 1 Molecular Diagnostic Laboratory, Johns Hopkins Aramco Healthcare, Dhahran 31311, Saudi Arabia; 2 College of Medicine, Alfaisal University, Riyadh 11533, Saudi Arabia; 3 Department of Public Health and Nutrition, The University of Haripur, Haripur 22610, Pakistan; 4 Department of Microbiology, Kogi State (Prince Abubakar Audu) University, Anyigba 10008, Nigeria; bello.k@ksu.edu.ng; 5 Medical Laboratory Department, Mohammed Al-Mana College for Medical Sciences, Dammam 34222, Saudi Arabia; zahedaa@machs.edu.sa; 6 Clinical Pharmacy Department, King Saud Medical City, Riyadh 11362, Saudi Arabia; amal.t@ksmc.med.sa; 7 Department of Pharmacy Practice, College of Pharmacy, Princess Nourah bint Abdulrahman University, Riyadh 11671, Saudi Arabia; haalrasheed@pnu.edu.sa; 8 Infectious Diseases Unit, Department of Medicine, King Faisal Specialist Hospital and Research Center, Riyadh 11564, Saudi Arabia; jalotaibi97@kfshrc.edu.sa; 9 Department of Infectious Disease, King Abdullah Medical Complex, Jeddah 6725, Saudi Arabia; drshoosho86@hotmail.com; 10 Gastroenterology Department, King Fahad Armed Forces Hospital, Jeddah 23831, Saudi Arabia; azaidan@kfafh.med.sa; 11 Department of Community Medicine and Health Care for Pilgrims, Faculty of Medicine, Umm Al-Qura University, Makkah 21955, Saudi Arabia; magarout@uqu.edu.sa; 12 Pediatric Infectious Diseases Unit, Pediatric Department, King Abdulaziz Hospital, Jeddah 23831, Saudi Arabia; tzaidan@moh.gov.sa; 13 Infection and Control Department, Armed Forces Hospital, Azaibah 130, Oman; highmoon1@hotmail.com; 14 Diagnostic Virology Laboratory, Maternity and Children Hospital, Eastern Health Cluster, Dammam 32253, Saudi Arabia; drsanaalshaikh@gmail.com; 15 Nursing Department of Vaccine Clinic, Hospital: Al Jamaeen Primary Health Care, Dammam 32467, Saudi Arabia; khalalawi@moh.gov.sa; 16 Department of Medicine, Royal College of Surgeons, D02 YN77 Dublin, Ireland; razialalqam@rcsi.com; 17 Department of Genetics Research, Institute for Research and Medical Consultations (IRMC), Imam Abdulrahman Bin Faisal University, Dammam 34221, Saudi Arabia; htoglu@iau.edu.sa; 18 Infection Prevention and Control Centre of Excellence, Prince Sultan Medical Military City, Riyadh 12233, Saudi Arabia; 19 Preventive and Community Medicine Department, Faculty of Medicine, University of Sousse, Sousse 4002, Tunisia

**Keywords:** Hepatitis B, Hepatitis C, drug users, prevalence, systematic review, meta-analysis

## Abstract

Hepatitis B virus (HBV) and Hepatitis C virus (HCV) contribute significantly to morbidity and mortality among drug users in Asia. This study systematically reviews and analyzes the pooled prevalence of HBV and HCV, considering geographic and methodological variations. A meta-analysis following PRISMA guidelines included data from PubMed, Scopus, and Google Scholar on studies on HBV or HCV or a combination of both within Asia. A random-effects model estimated pooled prevalence, with subgroup analyses by region, study design, diagnostic method, and publication year. A total of 112 studies were analyzed. The pooled HBV prevalence among drug users was 14.3% (95% CI: 11.5–17.6), highest in Malaysia (28.7%) and Vietnam (26.6%). HCV prevalence was 58.6% (95% CI: 54.0–63.0), with the highest rates in Vietnam (63.5%) and China (62.9%). Retrospective studies reported a higher prevalence than cross-sectional ones. The use of ELISA for initial screening followed up by PCR reduced heterogeneity, improving diagnostic accuracy. HBV prevalence declined after 2010, while HCV rates remained persistently high. The high burden of HBV and HCV among drug users in Asia underscores an urgent public health concern. Targeted interventions, including vaccination, harm reduction strategies, and improved access to antiviral treatments, are essential to curbing transmission and enhancing health outcomes.

## 1. Background

The hepatitis B virus and hepatitis C virus are major causes of chronic liver disease, cirrhosis, and hepatocellular cancer worldwide [1,2]. Despite the large advances in the prevention, treatment, and management approach for both viruses, they remain a high cause of public health concern for all countries [3]. HBV and HCV are great contributors to morbidity and mortality in highly endemic areas [4]. According to the WHO, there are almost 2 billion infected people with HBV worldwide, of which 257 million are chronic carriers, and it is responsible for approximately 887,000 deaths annually from related diseases [5]. HCV affects an estimated 58 million people worldwide, with 1.5 million new infections occurring annually and approximately 290,000 deaths occurring annually due to cirrhosis and liver cancer. This burden is disproportionately situated on the Asia continent [6].

The region harbors some of the world’s highest prevalence rates of HBV and HCV-in fact, as high as 10% of the population in some East and Southeast Asian nations are carriers of chronic HBV infections [7]. Similarly, HCV rates remain dangerously high for strata of the population that are at risk, drug users. That notwithstanding, despite public health efforts to promote vaccination programs for HBV and direct-acting antiviral treatments for HCV, which is an actual realistic way of tackling the infection, huge gaps in prevention and care persist notably among high-risk groups, especially PWUD [8,9].

The drug users, more particularly PWID, are a critical population that bears a high vulnerability to the infections of HBV and HCV [10]. The behavioral risk factors, such as needle sharing, interlink with the structural drivers’ lack of access to both harm reduction services and health care, thereby further elevating the vulnerability of this population [11]. In Asia, the high prevalence of injectable drug use is compounded by sociopolitical, cultural, and economic challenges that hinder optimal responses. Injecting drug use is a clearly recognized precipitating factor in the spread of both HBV and HCV [10,11].

Bloodborne pathogens, such as the aforementioned viruses, spread widely due to shared infected injecting equipment. This often is transmitted in settings lacking harm reduction programs, including NSP and OST [12].

Although HBV can be transmitted perinatally, sexually, or through unsanitary medical procedures, the intersection of injection drug use with poor harm reduction practices produces specific epidemiological risks [13]. By contrast, HCV is primarily transmitted through blood-to-blood contact, thereby positioning PWID as among the most heavily affected populations worldwide [14]. The epidemiology among drug users from HBV and HCV infection represents geographical, cultural, and economic diversity across Asia [15]. Southeast Asian countries such as Vietnam, Myanmar, and Cambodia have reported high prevalence rates of injectable drug use but face challenges of inadequate harm reduction infrastructure and limited healthcare access [16,17]. These conditions make for higher HBV and HCV prevalence among PWID in these countries [17]. In contrast, countries like Japan and the Republic of Korea in East Asia report lower incidence rates because of comprehensive public health programs, widespread HBV vaccination efforts, and strict pharmaceutical controls [17]. However, even within those countries, subgroups of IDUs with discrimination or legal persecution remain at significant risk of both infections [18].

Central Asia is another story. Political and economic turmoil in countries such as Kazakhstan, Kyrgyzstan, and Uzbekistan has left the public health structures very weak, and drug users are thus very vulnerable to the infections caused by HBV and HCV [19]. Narcotics trafficking and increasing injecting drug use in the region, in addition to a very low level of harm reduction programs, are adding to the problem.

Most of the legislation on drugs and policy enforcement has been punitive, which tends to force drug users underground and consequently limit their access to health care services and harm reduction measures [20]. Stigmatization and discrimination further marginalize this population and dissuade them from seeking immunization, testing, and treatment for viral hepatitis [21].

Harm reduction approaches, like NSPs, OST, and targeted HBV immunization programs, are the keys to reducing transmission risks [21]. However, the implementation of these programs varies considerably in Asia. Countries like Vietnam and Malaysia have introduced harm-reduction policies under their national public health programs. In contrast, others, such as the Philippines and Thailand, face serious barriers due to repressive drug laws [11,22].

The introduction of DAA revolutionized the treatment of HCV, but high cost and limited access are major challenges in most Asian countries. Despite the well-documented global burden of HBV and HCV, data on their prevalence among drug users in Asia remain fragmented and unreliable [22]. Many studies are focused on PWID and often use different methods, diagnostic criteria, and sampling methods, which make combining results and drawing meaningful conclusions from these studies quite problematic [23]. Most of the available literature is limited to country-specific studies with very little regional or subregional analysis to identify broader trends. Neonatal vaccination programs for HBV have drastically cut prevalence rates among the general population, but their effectiveness in high-risk groups of drug users is less certain [24]. Similarly, while breakthroughs in HCV treatment create reasonable grounds for optimism for eventual eradication, the lack of reliable prevalence and access to treatment data among PWID limits those efforts [25].

The smaller countries in the region and some understudied populations are significantly lagging, leaving policymakers with relatively scant information to develop targeted responses. Given the dual burden of HBV and HCV among drug users in the Asian setting, the need for a comprehensive review and meta-analysis cannot be understated, given the deficiency of these analyses in existing literature. The present study commits to an in-depth synthesis of current data pertaining to the prevalence of HBV and HCV in the region.

## 2. Methods

### 2.1. Research Design

The systematic review and meta-analysis were performed based on the Preferred Reporting Items for Systematic Reviews and meta-analysis criteria. According to the PRISMA criteria, this process ensures clarity, reproducibility, and comprehensiveness of the process for synthesizing available evidence [26]. Accordingly, the objectives of this review are to assess the incidence of HBV and HCV among drug users in Asia by highlighting geographical disparities in terms of the prevalence of the virus and critical gaps in the existing literature.

### 2.2. Eligibility Criteria

Studies were chosen based on the pre-defined inclusion and exclusion criteria of the study. These are:

Inclusion Criteria Investigation of drug users, whether injecting or not, in Asian countries. Investigations that report the prevalence of HBV and HCV as a point estimate or percentage, studies reporting HBV and HCV as co-infected with other diseases such as Human immunodeficiency virus (HIV). Cross-sectional studies, cohort, and case-control studies ensure the integrity of data is assured and published to date.

Exclusion Criteria Studies targeting a population unrelated to drug use or without specific prevalence figures for drug users. Systematic reviews, editorials, conference paper presentations, and opinions that do not present original data. Studies with a lack of uncertain information on the prevalence of HBV and HCV. Duplicate publication or overlapping datasets.

### 2.3. Information Sources

Literature was retrieved from the following electronic databases: PubMed, Google Scholar, Scopus, Web of Science, and Science Direct. Grey literature sources were explored for government reports and institutional repositories to reduce publication bias. Reference lists from eligible publications were further scanned for additional studies.

### 2.4. Search Strategy

The search strategy combines MeSH terminology with free-text keywords in order to ensure a comprehensive search; there was no language restriction in the search strategy. Examples of terminology include:

Hepatitis B: (“Hepatitis B”, “HBV”, “Hepatitis B Virus infection.”), Hepatitis C: (“Hepatitis B”, “HCV”, “Hepatitis C Virus infection.”) Substance users: “Substance use”, “Drug addiction”, “Injection drug users”, “People who inject drugs (PWID)”. Asia: Specific country names and “Asia” as a continent/region. To restrict the search output, Boolean operators AND, OR, and NOT were applied. Truncation and wild card symbols were also used to capture changing terminology, for example, “drug use” and “Asia”. The search strategy was peer-reviewed for completeness and accuracy, and details about the search strategy are provided in Supplementary File S1.

### 2.5. Selection of Studies

The selection was performed according to a two-track approach: Two independent reviewers reviewed the titles and the abstracts of the retrieved materials for possibly relevant articles. The third reviewer solved the disagreement. All eligible articles identified during preliminary screening entered a detailed full-text evaluation based on the set inclusion and exclusion criteria. A PRISMA flow diagram was used to record the number of studies identified, screened, excluded, and included at each step.

### 2.6. Data Extraction

Data were extracted independently by two reviewers using a predesigned standardized data collection instrument. The following information was extracted:

Study Characteristics: Author(s), Year of publication, Country of study, and Study design. Participants’ Characteristics: Sample size demographic data, such as age, sex, and type of drug use-injecting or non-injecting. Performance Indicators: HBV prevalence, confidence interval, if available, and diagnostics used, such as HBsAg tests, etc. Risk Factors: Factors associated with HBV and HCV prevalence, if any, Quality Factors: Response rate, Sampling method, and Study limitations. Any disagreements that occurred in data extraction were resolved by consensus or with the help of a third reviewer.

### 2.7. Risk of Bias Assessment

The risk of bias in the selected studies was measured using a validated tool, namely the Joanna Briggs Institute Critical Appraisal Checklist for Prevalence studies [27]. Details of the quality checklist for the studies are provided in Supplementary File S2. The domains evaluated included Sampling strategy, representativeness of sample, validity, and reliability of HBV diagnostic method, and handling of missing data. The risk of bias in each study was assessed as low, moderate, or high. Sensitivity analyses were conducted on the basis of excluding studies with a high risk of bias.

### 2.8. Statistical Analysis

The prevalence data from the included studies were pooled using a random-effects meta-analysis model, considering the heterogeneity between studies. To determine heterogeneity, the I^2^ statistic was used, and thresholds of 25%, 50%, and 75% showed low, moderate, and high heterogeneity, respectively [28,29]. Subgroup analyses were conducted by exploring potential sources of heterogeneity according to the following: country or subregion in Asia, study setting-community-based versus institution-based Year of publication. We evaluated publication bias with funnel plots and Egger’s test [30]. If asymmetry was observed, pooled estimates were adjusted using the trim-and-fill method [30].

## 3. Result

### 3.1. Study Selection

A comprehensive search of six electronic databases produced a total of 5164 records. Following the elimination of duplicates, 911 distinct articles persisted for additional evaluation. A meticulous analysis of the titles and abstracts of these publications led to the elimination of 3966 articles that failed to satisfy the established inclusion criteria. The main grounds for exclusion at this stage were irrelevance to the study topic, No prevalence information for HBV/HCV, or insufficient data on HBV/HCV prevalence.

After the preliminary screening, the entire text of the remaining articles was scrutinized to evaluate their eligibility for inclusion in the review. The comprehensive assessment resulted in the deletion of an additional 607 papers due to issues including data duplication, lack of HBV/HCV prevalence data, and incomplete or unclear prevalence reporting. In total, 112 publications satisfied all inclusion criteria and were considered appropriate for qualitative synthesis and meta-analysis.

**Figure 1:**
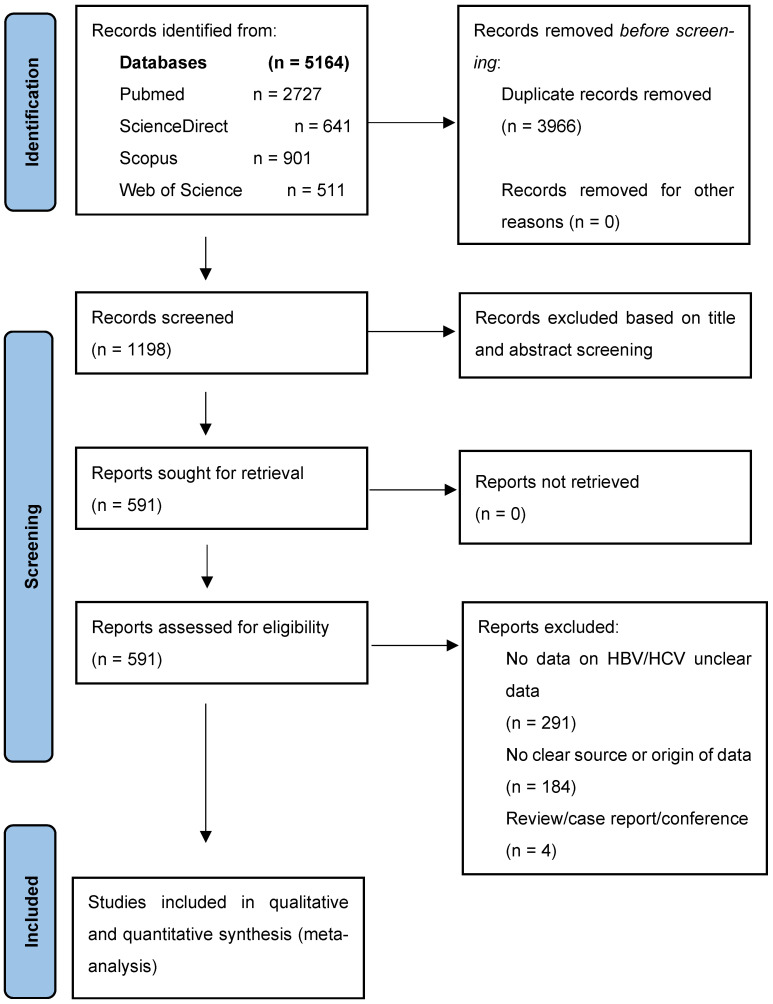
Summary of the studies selection and screening process.

Figure 1 visually summarizes the research selection process, detailing each stage of article identification, screening, and inclusion, as well as the rationale for rejection at each step. The stringent selection method guaranteed the incorporation of high-caliber research that corresponded with the aims of this systematic review and meta-analysis.

### 3.2. Characteristics of the Included Studies

Table 1 delineates the attributes of diverse studies on Hepatitis B Virus (HBV) and Hepatitis C Virus (HCV) infections carried out in various countries, predominantly in Asia. These studies collectively offer an extensive overview of HBV and HCV prevalence among various populations, utilizing a combination of cross-sectional and retrospective methodologies, with detection techniques primarily reliant on ELISA (Enzyme-Linked Immunosorbent Assay) and PCR (Polymerase Chain Reaction). The results indicate substantial discrepancies in infection rates, underscoring the public health challenges presented by these viruses, especially in the Asia-Pacific area.

The Hepatitis B Virus (HBV) has been documented in numerous research studies, with sample sizes varying from 34 to 7740 persons. In Vietnam, extensive investigations like those conducted by Barnaby and others surveyed 7740 persons and identified 1321 positive cases, indicating a substantial disease burden. In a similar vein, the research conducted by Nicholas and others, which encompassed 1444 participants, identified 875 cases of HBV positivity. The elevated prevalence corresponds with Vietnam’s classification as a high-endemic area for HBV. In contrast, smaller-scale studies such as those by Ishizak and others offer nuanced insights into certain cohorts, with differing positive rates indicating potential demographic or behavioral risk factors affecting HBV transmission.

The prevalence of HBV in nations such as Thailand and Malaysia is a significant part of regional health issues. Verachai and others reported 124 HBV-positive cases from a sample of 303 persons in Thailand, whereas Akthar and others identified 86 positive cases among 664 samples in Malaysia. These statistics highlight the significant impact of HBV, especially in smaller populations, requiring public health initiatives customized to local epidemiological trends. Studies on the Hepatitis C Virus (HCV) demonstrate significant diversity in prevalence rates. Dunford et al. and others found a concerning positive rate of 1000 out of 1434 persons sampled in Vietnam, underscoring the urgent necessity for focused HCV interventions in the area. Conversely, research conducted in Iran, including that of Hsieh and others, identified 513 HCV-positive cases among 562 samples, reflecting a comparably significant burden. The research conducted by Barnaby and others in Vietnam, using a substantial sample of 5461 participants, identified 3157 cases of HCV positivity, highlighting the concurrent prevalence of HBV and HCV in specific groups. India exhibits varied results in the prevalence of HBV and HCV across multiple research projects. McFall and others documented 1901 HCV-positive cases out of 6457 samples, underscoring substantial disease prevalence in one of the most populous countries globally. Comparable research conducted by Solomon and others revealed consistent HCV prevalence, with 293 and 566 positive cases, respectively, across intermediate sample sizes. These findings underscore the necessity for comprehensive public health initiatives to alleviate the HCV burden in India. The research methods predominantly employ Enzyme-Linked Immunosorbent Assay (ELISA) at the first level of screening, with some also employing Polymerase Chain Reaction (PCR) as confirmation. This also suggests the need for standardized diagnostic kits to provide accuracy and comparability of the data across sites. ELISA’s affordability and ease of use have made it pivotal in mass-scale screening, particularly in resource-poor areas. Despite that, PCR research offers the greater sensitivity and specificity required for case confirmation and understanding viral load dynamics.

In addition to geographical and methodological variations, the temporal aspect of this research is significant. Data extending across several decades, from 1994 (Li and others China) to 2023 (Nicholas and others Vietnam), demonstrate trends in the prevalence of HBV and HCV. For example, although certain earlier studies from China indicate moderate prevalence of HBV and HCV, more recent data from Vietnam demonstrate consistently elevated rates. This temporal study indicates either persistent transmission dynamics or the influence of improved screening and diagnostic capabilities in recent years.

These investigations also notably reveal regional patterns. Southeast Asia, exemplified by Vietnam, Thailand, and Malaysia, demonstrates some of the highest prevalence rates of HBV and HCV. In Vietnam, numerous research studies continuously indicate disturbingly elevated positive rates, with Barnaby and others and Nicholas and others highlighting the substantial disease burden in the country. Likewise, research conducted in Taiwan, including that by Hsieh and others, reveals a significant prevalence of HCV, with 87 positive cases identified among 566 samples, underscoring another region of considerable burden.

Conversely, the Middle Eastern setting, illustrated by research from Iran, demonstrates diverse incidence rates of HBV and HCV. Abbasali and others identified 65 HBV-positive patients from 539 samples, whereas Davoodian and others documented 196 HCV-positive cases from 249 samples. The data indicate localized epidemiological factors affecting HBV and HCV prevalence in the region.

The public health ramifications of these discoveries are substantial. The elevated prevalence rates of HBV and HCV in numerous countries emphasize the critical necessity for improved vaccine initiatives (for HBV), public awareness efforts, and effective antiviral treatment approaches. The concurrent prevalence of HBV and HCV in places like Vietnam and India necessitates integrated strategies to manage both illnesses simultaneously. Moreover, the influence of socioeconomic factors, healthcare infrastructure, and behavioral determinants on these incidence patterns necessitates additional examination.

### 3.3. Pooled Prevalence of HBV Among Drug Users

**Figure 2:**
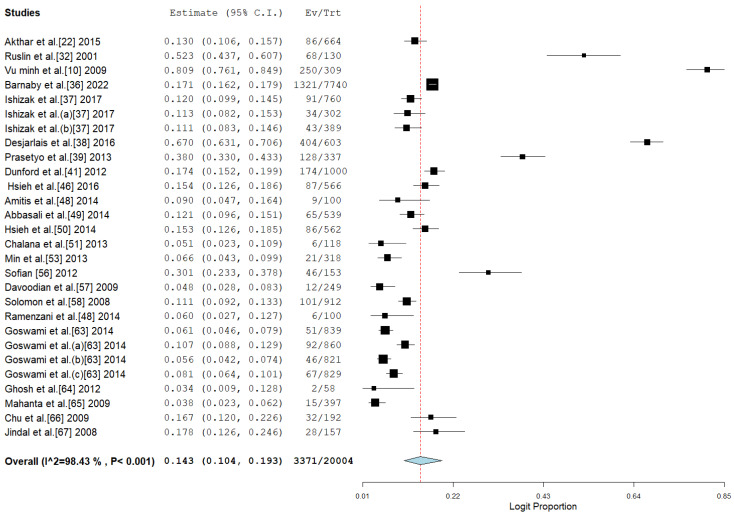
Forest plot showing the pooled prevalence of HBV among drug users in Asia [10,22,32,36,37,38,39,41,46,48,49,50,51,53,56,57,58,63,64,65,66,67].

**Figure 3:**
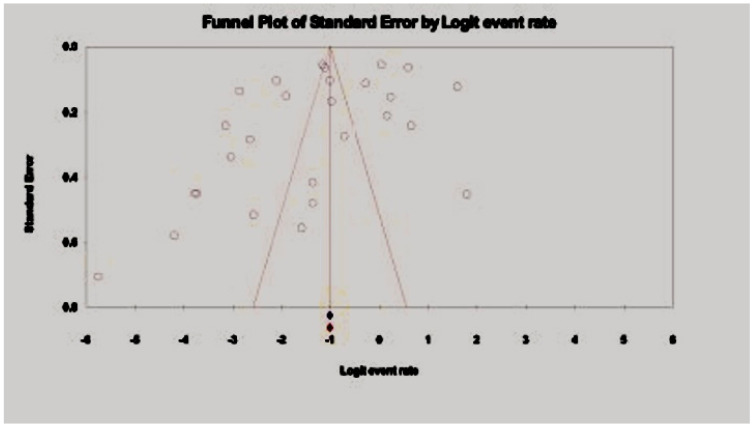
Funnel plot showing publication bias of the pooled prevalence of HBV among drug users in Asia. Egger’s *p* = 0.0145.

The study offers an in-depth examination of the pooled prevalence of hepatitis B virus (HBV) among drug users in Asia, with significant results depicted in the forest plot (Figure 2) and indications of possible publication bias shown in the funnel plot (Figure 3). The aggregated prevalence estimates provide valuable insights into the impact of HBV within this high-risk group.

The forest plot highlights significant variability among the analyzed studies, indicating variations in study environments, participant groups, and research methods. The significant variability indicates that the prevalence of HBV is not evenly spread throughout the region but rather shaped by specific local epidemiological and social influences. The pooled prevalence acts as a crucial summary measure for estimating the regional burden while also recognizing this variability.

The value of *p* = 0.0145 obtained from the funnel plot analysis indicates the presence of publication bias in the included studies, as illustrated in Figure 3.

### 3.4. Pooled Prevalence of HCV Among Drug Users in Asia

**Figure 4:**
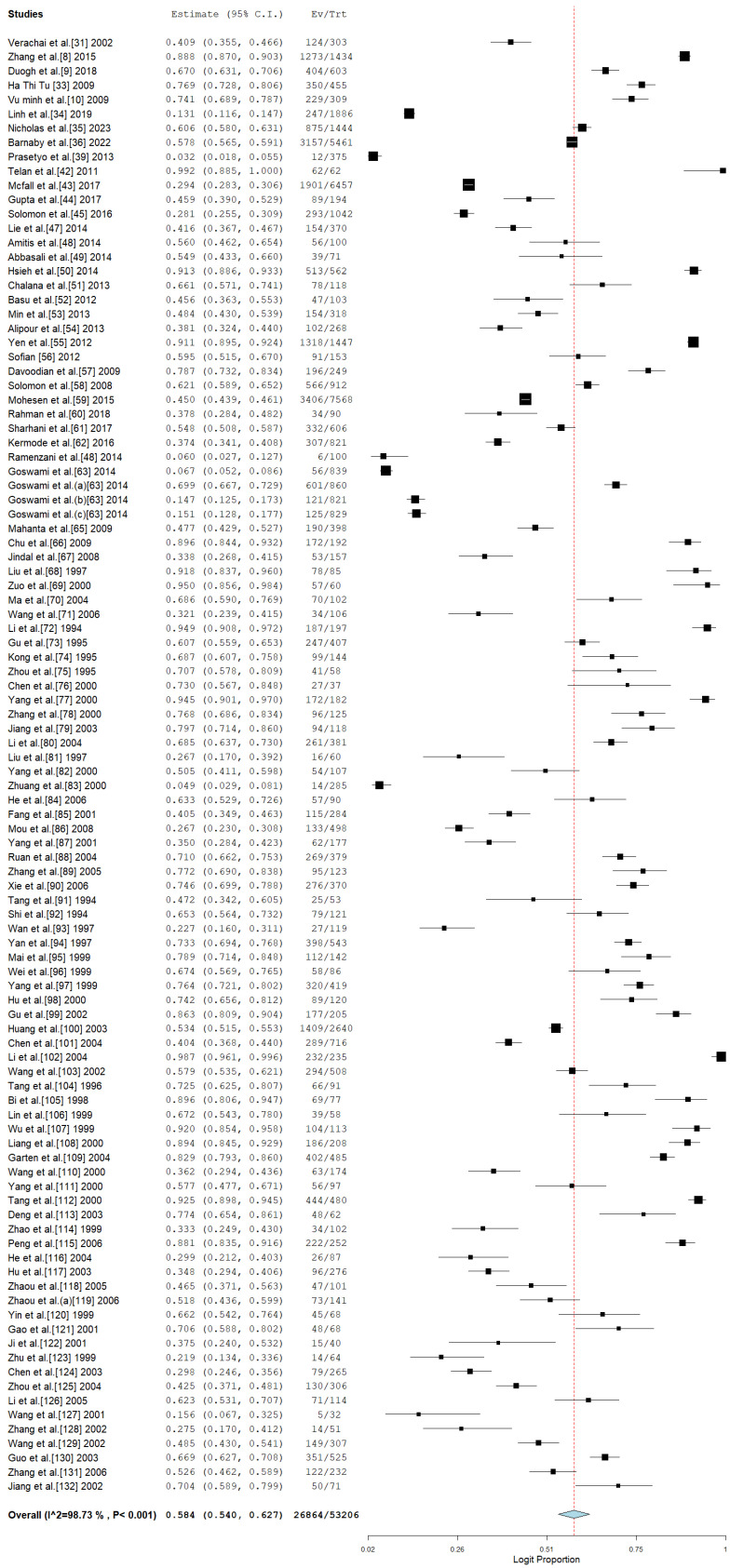
Pooled prevalence of HCV among drug users in Asia [8,9,10,31,33,34,35,36,39,42,43,44,45,47,48,49,50,51,52,53,54,55,56,57,58,59,60,61,62,63,64,65,66,67,68,69,70,71,72,73,74,75,76,77,78,79,80,81,82,83,84,85,86,87,88,89,90,91,92,93,94,95,96,97,98,99,100,101,102,103,104,105,106,107,108,109,110,111,112,113,114,115,116,117,118,119,120,121,122,123,124,125,126,127,128,129,130,131,132].

**Figure 5:**
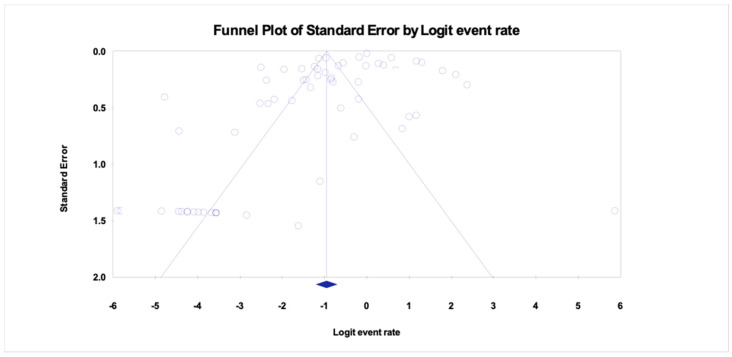
Funnel plot of HCV among drug users in Asia. Egger’s *p* = 0.136.

The analysis presented delivers a thorough assessment of the pooled prevalence of hepatitis C virus (HCV) among drug users in Asia. Figure 4 showcases the pooled prevalence via a forest plot, while Figure 5 illustrates the funnel plot to evaluate publication bias. The pooled prevalence serves as a crucial epidemiological metric for assessing the burden of HCV in a population that is especially susceptible due to behaviors linked to drug use, including needle sharing.

The combined prevalence estimate represents an aggregation of various studies and underscores the considerable impact of HCV within the drug-using population. The latter highlights the essential requirement for focused public health initiatives. Nonetheless, as shown by Egger’s *p* = 0.136, there is no substantial evidence of publication bias in the studies that were included.

### 3.5. Subgroup Meta-Analysis

#### 3.5.1. HBV Subgroup

**Figure 6:**
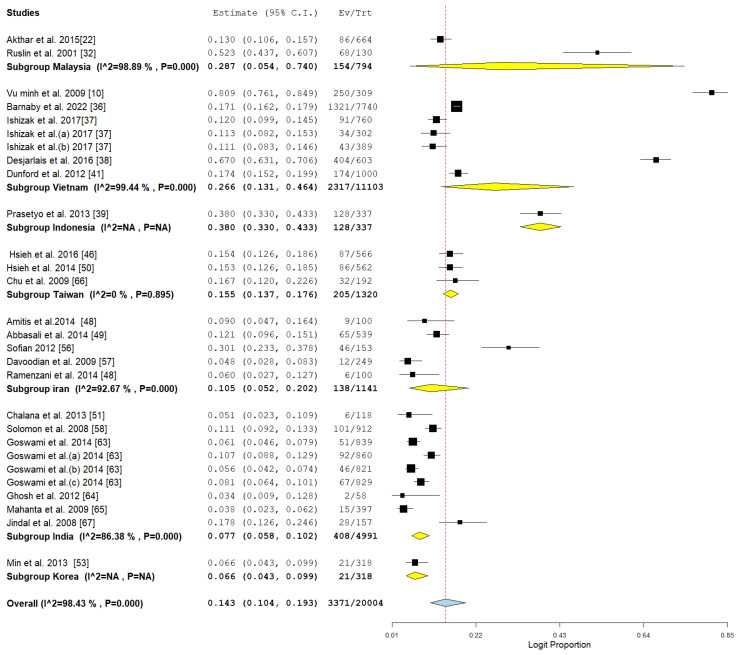
Subgroup meta-analysis of the prevalence of HBV among drug users in ASIA in relation to country [10,22,32,36,37,38,39,46,48,49,50,51,56,57,58,63,64,65,66,67].

This meta-analysis examines the prevalence of Hepatitis B Virus (HBV) among drug users in Asia, categorized by various parameters such as country, study design, detection method, and publication year. The data reveal variations in geography and methodology, illustrating the changing patterns of HBV infection over time, as shown in Table 2. The findings indicate significant differences in HBV prevalence among various Asian nations. Malaysia exhibited the highest prevalence among the countries reported (28.7%, CI: 5.4–74.0), although this was derived from only two studies, indicating considerable heterogeneity. Vietnam came next with a prevalence of 26.6% (CI: 13.1–46.4), backed by seven studies that also demonstrated significant heterogeneity. Countries exhibiting notable prevalence rates comprise Indonesia (26.6%, based on a single study), Taiwan (15.5%, CI: 13.7–17.6), and Iran (10.5%, CI: 5.2–20.2), indicating considerable heterogeneity. Korea (6.6%, single study) and India (7.7%, CI: 5.8–10.2) exhibited relatively low HBV prevalence among drug users, as illustrated in Table 2 and Figure 6.

**Figure 7:**
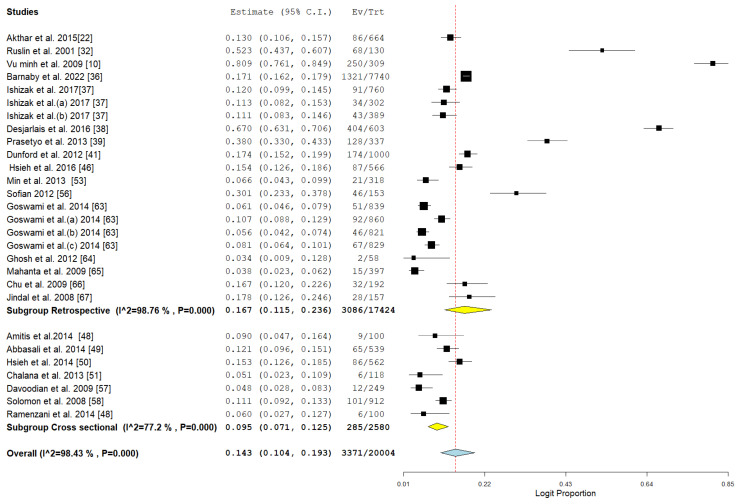
Subgroup meta-analysis of the pooled prevalence of HBV among drug users in Asia in relation to study design [10,22,32,36,37,38,39,41,46,48,49,53,56,57,58,63,64,65,66,67].

The prevalence of HBV was observed to be greater in retrospective studies (16.7%, CI: 11.5–23.6, I^2^ = 98.76%, *p* < 0.001) in comparison to cross-sectional studies (9.5%, CI: 7.1–12.5, I^2^ = 77.2%, *p* < 0.001). This difference may stem from variations in data collection techniques and the likelihood of selection bias in retrospective designs, as illustrated in Figure 7.

**Figure 8:**
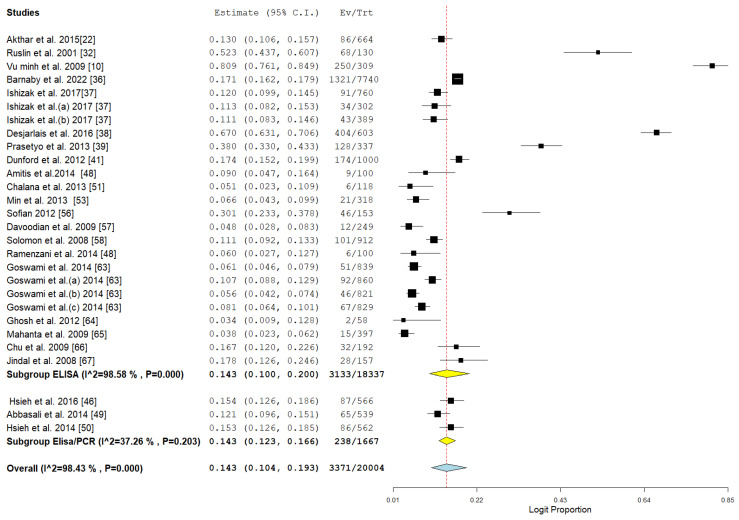
Subgroup meta-analysis of the pooled prevalence of HBV among drug users in Asia in relation to method of detection [10,22,32,36,37,38,39,41,48,49,50,51,53,56,57,58,63,64,65,66,67].

HBV detection through ELISA (25 studies) indicated a pooled prevalence of 14.3%, accompanied by significant heterogeneity. In contrast, the combination of ELISA and PCR (three studies) produced the same prevalence but exhibited much lower heterogeneity, as illustrated in Figure 8. This suggests that the integration of detection methods could minimize variability and enhance diagnostic precision, as illustrated in Table 2 and Figure 8.

**Figure 9:**
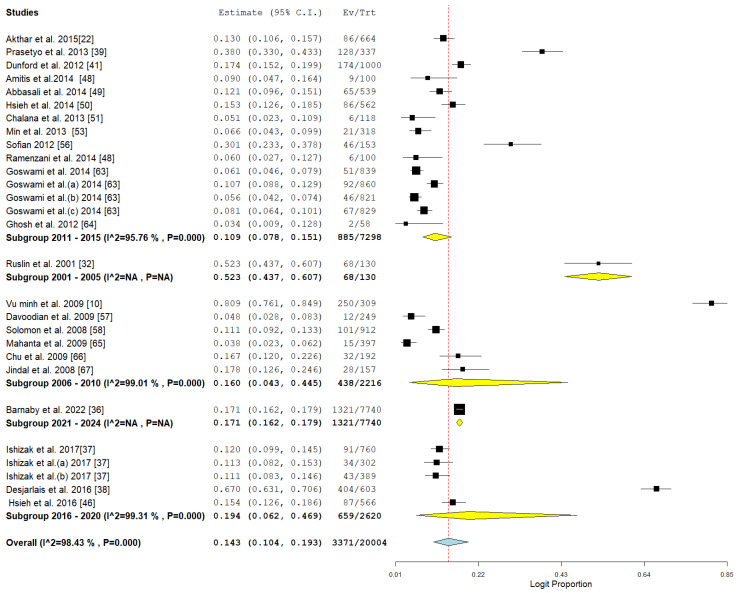
Subgroup meta-analysis of the pooled prevalence of HBV among drug users in Asia in relation to year of publications [10,22,32,36,37,38,39,41,46,48,49,50,51,53,57,58,63,64,65,66,67].

There were clear temporal trends observed in the prevalence of HBV among individuals who use drugs. The peak prevalence observed was 52.3% (CI: 43.7–60.7) during the period of 2001–2005, as indicated by a singular study, after which there was a notable decrease to 16% in the subsequent years of 2006–2010. Nonetheless, a notable increase was recorded in later intervals: 19.4% (CI: 6.2–46.9, during 2016–2020) and 17.1% (CI: 16.2–17.9) from 2021 to 2024, highlighting ongoing public health issues, as illustrated in Table 2 and Figure 9.

#### 3.5.2. HCV Subgroup

The subgroup analysis shown in Table 3 emphasizes the aggregated prevalence of hepatitis C virus (HCV) among drug users across various countries, study methodologies, detection techniques, and years of publication. The results offer a detailed insight into the fluctuations in HCV prevalence shaped by these factors. The data exhibit considerable variability across the majority of subgroups.

**Figure 10:**
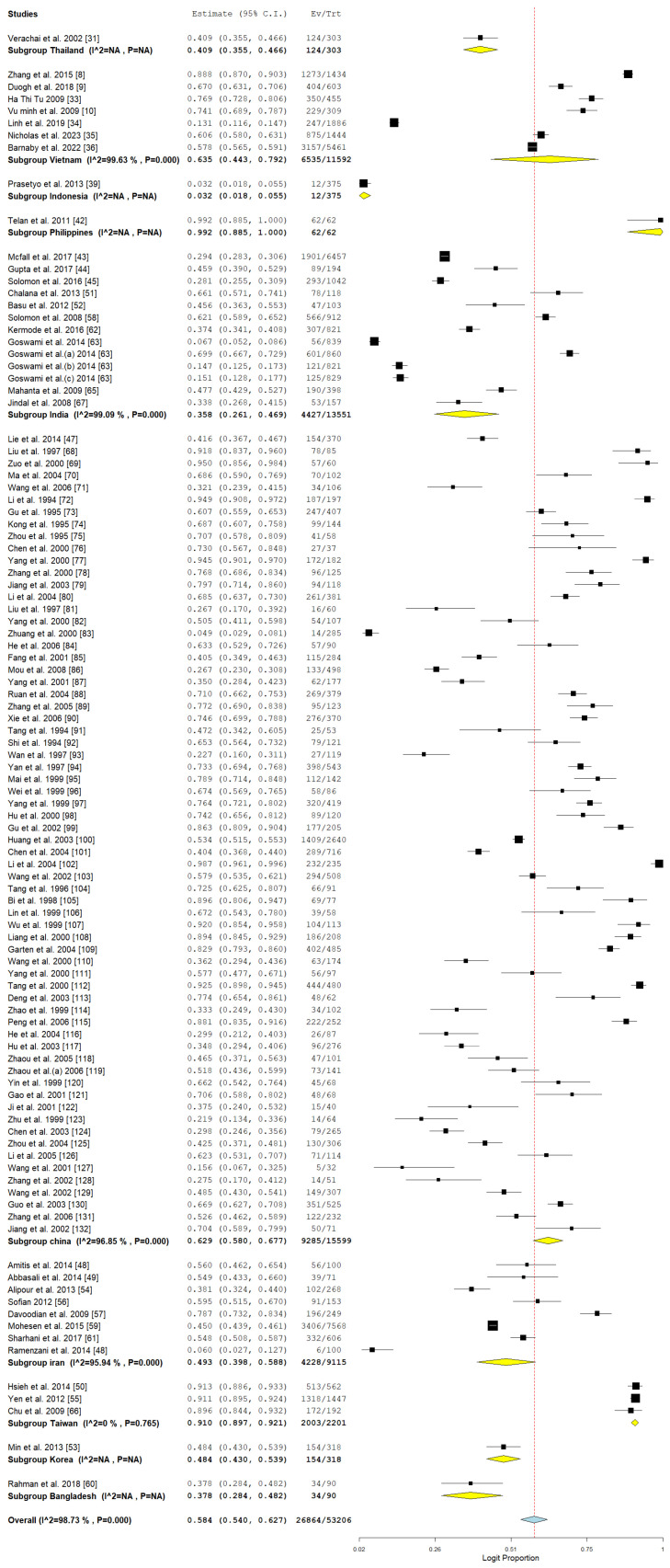
Subgroup pooled prevalence of HCV among drug users in Asia inrelation to country [8,9,10,31,32,33,34,35,36,39,42,43,44,45,47,48,49,50,51,52,53,54,55,56,57,58,59,60,61,62,63,64,65,66,67,68,69,70,71,72,73,74,75,76,77,78,79,80,81,82,83,84,85,86,87,88,89,90,91,92,93,94,95,96,97,98,99,100,101,102,103,104,105,106,107,108,109,110,111,112,113,114,115,116,117,118,119,120,121,122,123,124,125,126,127,128,129,130,131,132].

The variation in HBV prevalence across different countries indicates significant regional disparities in transmission dynamics and healthcare systems. The prevalence in Vietnam was notably high at 63.5% (95% CI: 44.3–79.2) (Figure 10), with significant heterogeneity observed. In a similar vein, China exhibited a notably high prevalence of 62.9% (95% CI: 58.0–67.7), highlighting the endemic nature in Southeast Asia. In contrast, the Philippines exhibited an extraordinarily high prevalence (99.2%, 95% CI: 88.5–100.0), indicating distinct epidemiological or sampling circumstances in that environment. Iran indicated a prevalence of 49.3% (95% CI: 39.8–58.8) with significant heterogeneity, whereas India exhibited a prevalence of 35.8% (95% CI: 26.1–46.9), as illustrated in Figure 10. The results indicate significant challenges throughout South and Southwest Asia. Taiwan, through the aggregation of three studies, demonstrated a prevalence of 91% (95% CI: 89.7–92.1) and exhibited low heterogeneity, suggesting a consistent HBV burden and possibly efficient screening or reporting systems.

**Figure 11:**
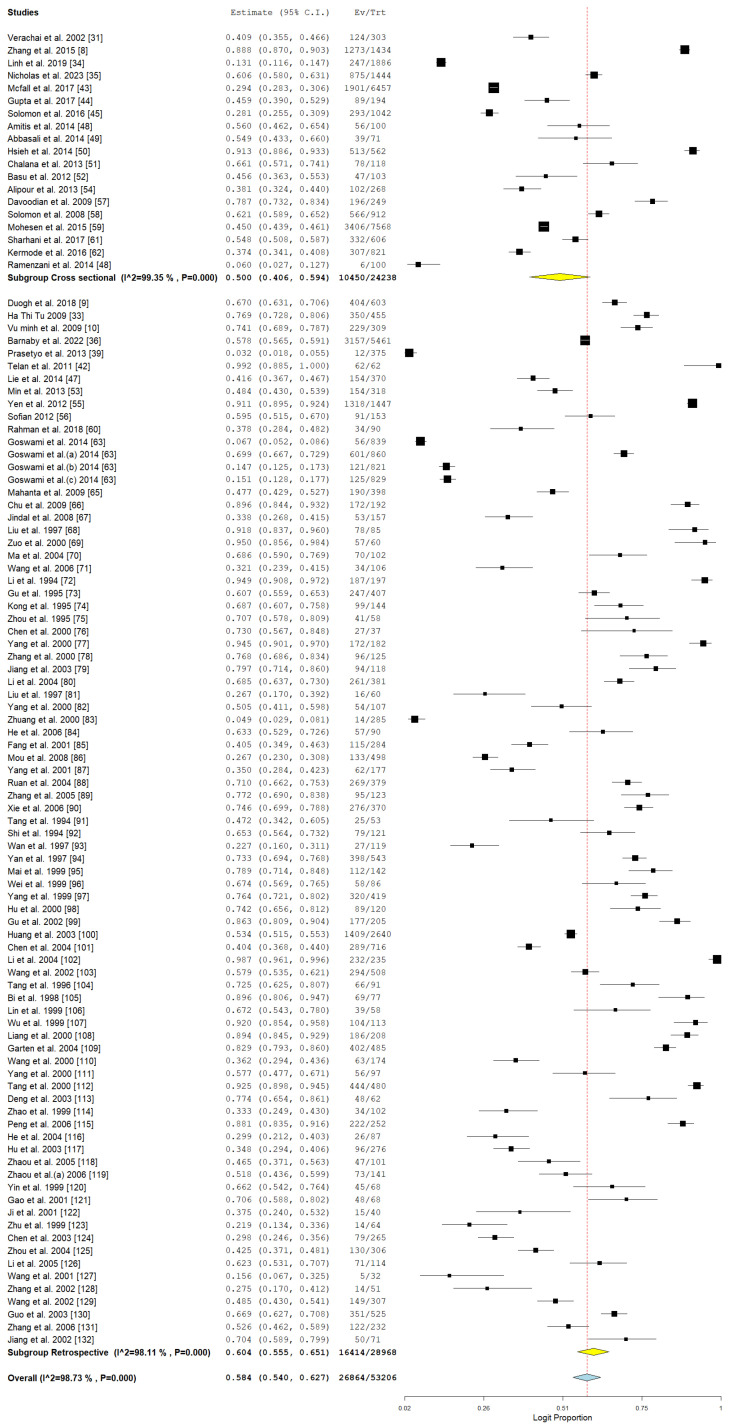
Subgroup meta-analysis of the pooled prevalence of HCV among drug users in relation to study designs [9,10,31,33,34,35,36,39,42,43,44,47,48,49,50,51,52,53,54,55,56,57,58,59,60,61,62,63,65,66,67,68,69,70,71,72,73,74,75,76,77,78,79,80,81,82,83,84,85,86,87,88,89,90,91,92,93,94,95,96,97,98,99,100,101,102,103,104,105,106,107,108,109,110,111,112,113,114,115,116,117,118,119,120,121,122,123,124,125,126,127,128,129,130,131,132].

The design of the studies had a significant impact on prevalence estimates. Retrospective studies indicated a higher pooled prevalence (60.4%, 95% CI: 55.5–65.1) in contrast to cross-sectional studies (50%, 95% CI: 40.6–59.4), as illustrated in Figure 11. The significant variability observed in both retrospective and cross-sectional studies highlights the disparities in methodologies, sampled populations, and contextual factors.

The method used for HBV detection has impacted the reported prevalence rates. Research utilizing enzyme-linked immunosorbent assay (ELISA) indicated a pooled prevalence of 58.6% (95% CI: 54.0–63.0), accompanied by significant heterogeneity, as illustrated in Table 3. Studies utilizing polymerase chain reaction (PCR) indicated a lower prevalence of 44.4% (95% CI: 23.1–68.0), whereas studies that combined ELISA and PCR demonstrated the highest prevalence at 67.4% (95% CI: 24.8–92.9). The variability highlights the importance of methodological sensitivity and specificity in establishing prevalence.

**Figure 12:**
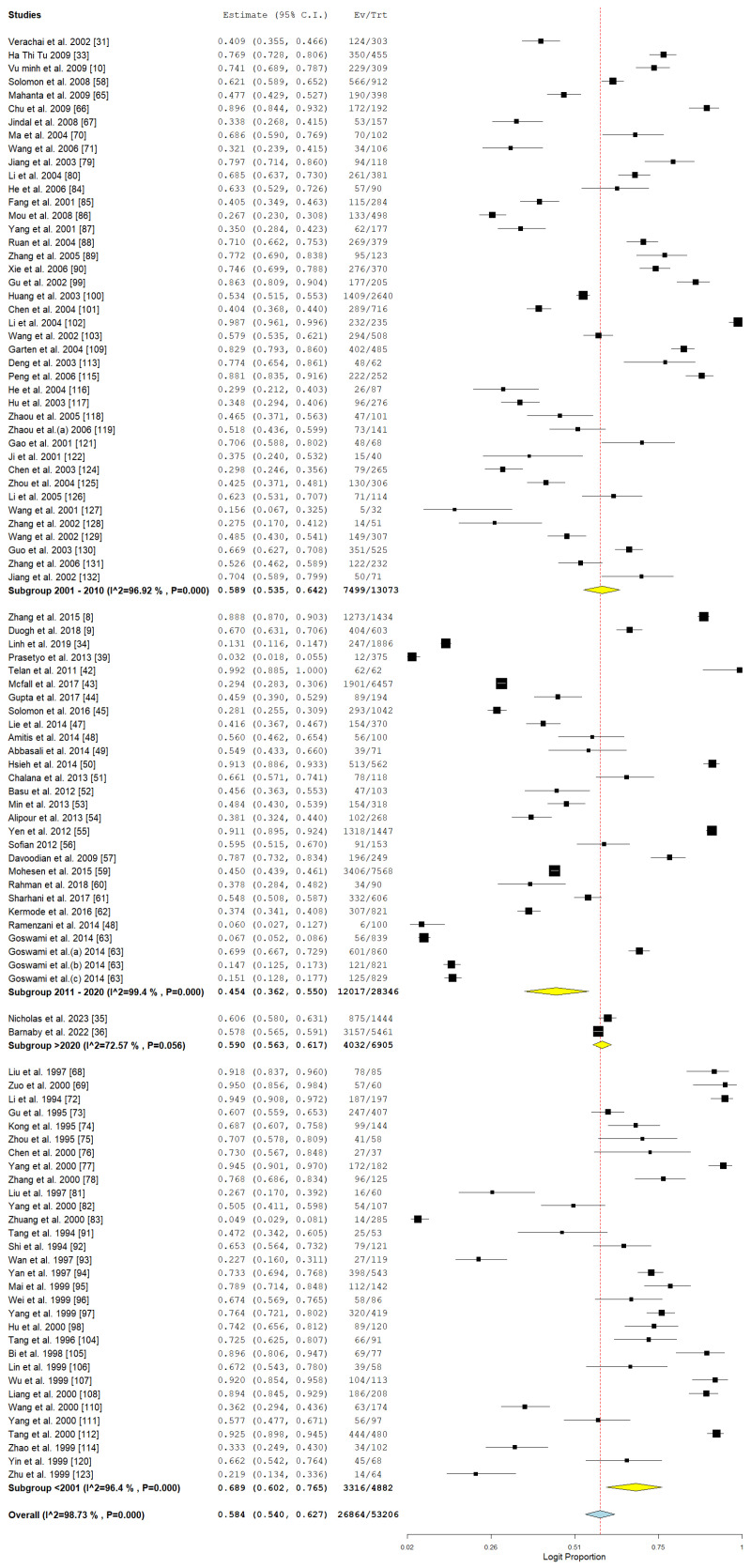
Subgroup meta-analysis of the pooled prevalence of HCV among drug users in relation to year of publication [8,9,10,31,33,34,39,42,43,44,45,47,48,49,50,51,52,53,54,55,56,57,58,59,60,61,62,63,70,71,79,80,81,82,83,84,85,86,87,88,89,90,91,92,93,94,95,96,97,98,99,100,101,102,103,104,105,106,107,108,109,110,111,112,113,114,115,116,117,118,119,120,121,122,123,124,125,126,127,128,129,130,131,132].

The analysis of the temporal distribution of studies indicated that earlier research (prior to 2001) revealed a higher pooled prevalence of 65.6% (95% CI: 54.1–77.2), whereas studies conducted between 2001 and 2010 showed a prevalence of 57.1% (95% CI: 49.7–64.5), as illustrated in Figure 12. Studies conducted between 2011 and 2020 indicated a noteworthy reduction in prevalence, recorded at 47.6% with a 95% confidence interval of 35.3 to 59.9. The data indicate a positive trajectory in public health initiatives, vaccination rates, and harm reduction approaches as time progresses. Nevertheless, studies conducted after 2020 indicated an increase in prevalence (59%, 95% CI: 56.3–61.7), albeit based on a limited dataset (*n* = 2), which may suggest the influence of sampling or publication biases.

## 4. Discussion

The pooled prevalence of HBV and HCV among drug users in Asia highlights a significant public health concern [16], illustrating the intricate interactions of behavioral, structural, and systemic elements that increase the susceptibility of this group to infection [7]. Individuals who use drugs, especially those who engage in injection practices, encounter heightened risks for HBV and HCV transmission as a result of needle sharing, unprotected sexual behaviors, and exclusion from healthcare services [10].

The significant occurrence of HBV among individuals who use drugs aligns with findings that injecting drug use is a major factor in the spread of bloodborne viruses [10]. The ability of HBV to persist on surfaces and in dried blood for prolonged durations heightens the risk of transmission via contaminated injecting equipment [13]. The use of shared needles and paraphernalia establishes clear routes for HBV transmission, positioning drug users as one of the most vulnerable groups for this infection [133]. In a similar vein, the significant occurrence of HCV among individuals who use drugs can be linked to the virus’s exceptional durability and its main method of spread via blood-to-blood contact [6]. In contrast to HBV, HCV does not have a preventive vaccine, which significantly increases the disease burden for individuals involved in unsafe injecting practices [134].

Alongside the introduction of certain behaviors, the sexual transmission of HBV poses a considerable risk among individuals who use drugs. Engaging in high-risk sexual behaviors, such as unprotected intercourse and transactional sex, significantly increases their vulnerability to HBV infection [135]. Structural barriers, including stigma and discrimination, intensify these risks by restricting access to prevention, testing, and treatment opportunities [136,137]. Populations that use drugs and are marginalized frequently encounter social exclusion, limited access to healthcare services, and increased susceptibility to infectious diseases [137]. Confronting these structural challenges necessitates strategies that emphasize fairness, diminish stigma, and incorporate care services customized to the requirements of individuals who use drugs.

The geographic variability in HBV and HCV prevalence underscores significant regional disparities in disease epidemiology. Vietnam and Malaysia show the highest prevalence of HBV among drug users, highlighting deficiencies in healthcare infrastructure, inadequate vaccination initiatives, and restricted harm reduction strategies [22]. In a similar vein, Vietnam and China exhibit the highest prevalence rates of HCV, aligning with the understanding that these areas are endemic for bloodborne infections [6,10,138]. In contrast, nations such as Korea and India show relatively lower prevalence rates of HBV and HCV, likely due to superior healthcare infrastructure, broader vaccination coverage, and improved harm reduction strategies [139]. The observed disparities highlight the necessity of customizing interventions to respond to distinct regional contexts and healthcare issues effectively.

The variability seen in prevalence estimates reflects differences in public health policies, cultural perspectives on drug use, and the accessibility of preventive measures. In addition, nations that implement strong harm reduction strategies, including needle and syringe exchange programs and opioid substitution therapy, show decreased prevalence rates as a result of diminished chances for unsafe injecting behaviors. On the other hand, punitive strategies regarding drug use, which emphasize criminalization instead of harm reduction, intensify the disease burden by pushing drug use into secrecy and restricting access to healthcare services [140,141]. Areas with insufficient healthcare infrastructure encounter further difficulties, including a lack of diagnostic capabilities and limited access to antiviral therapies, which exacerbates the incidence of HBV and HCV among individuals who use drugs [141].

Socioeconomic disparities represent a crucial element influencing the elevated rates of HBV and HCV among drug-using populations. Individuals who use drugs frequently come from economically disadvantaged backgrounds, facing barriers to healthcare and preventive measures [142,143,144]. Financial obstacles hinder numerous individuals from obtaining HBV vaccination programs or expensive direct-acting antivirals (DAAs) for HCV, which are crucial for alleviating the disease burden. Furthermore, the cultural stigma surrounding drug use in numerous Asian societies acts as a barrier, preventing individuals from pursuing medical assistance or engaging in public health initiatives [136,137]. To tackle these disparities, it is essential to implement a comprehensive strategy that integrates financial assistance, enhanced access to healthcare, and active community involvement to reach those populations that are underserved effectively.

Publication bias has been identified as a significant issue in the examination of HBV prevalence; this bias probably arises from an increased tendency to publish studies that yield significant or positive results, which can lead to inflated pooled prevalence estimates and exaggerate the burden of the disease. Although the analysis of HCV prevalence showed less apparent publication bias, the heterogeneity among studies indicates variations in methodology and contextual factors [87]. The differences in study design, sample size, diagnostic methods, and population characteristics lead to this heterogeneity, making the pooled estimates more reliable.

The significance of diagnostic methods in establishing prevalence estimates is especially noteworthy. Investigations utilizing ELISA for the detection of HBV and HCV frequently indicated elevated prevalence rates, potentially shaped by the method’s cost-effectiveness and accessibility [24,145,146]. Nonetheless, the potential for false positives or negatives in ELISA, stemming from differing antigen concentrations, brings into question its diagnostic reliability [147]. PCR-based methods, although exhibiting greater sensitivity and specificity, are utilized less often because of their elevated costs and logistical demands [148]. The integration of ELISA and PCR methods resulted in decreased heterogeneity in prevalence estimates, highlighting the importance of utilizing various diagnostic techniques to improve accuracy and comparability.

The changes in HBV prevalence over time illustrate the effects of worldwide public health initiatives, particularly the implementation and expansion of HBV vaccination programs [149,150]. The noted decrease in prevalence following 2010 corresponds with increased vaccination efforts and the advocacy of harm reduction approaches. Nonetheless, the increase in prevalence observed in recent years may indicate difficulties in maintaining these initiatives, especially within marginalized groups like drug users [151]. Obstacles to vaccination and treatment adoption, such as stigma, limited awareness, and inadequate healthcare access, continue to be ongoing challenges [149,152]. The consistently high prevalence rates of HCV over time highlight the critical necessity for increased testing and improved access to direct-acting antivirals, which have transformed HCV treatment with cure rates surpassing 95%.

The consistently elevated rates of HBV and HCV among drug users in Asia highlight the critical necessity for focused public health strategies. Strategies aimed at harm reduction, including needle and syringe exchange programs and opioid substitution therapy, have shown effectiveness in decreasing the transmission of bloodborne infections [13]. It is essential to broaden these programs to encompass underserved areas and align them with wider public health strategies to reduce transmission risks effectively. It is essential to prioritize vaccination programs for HBV among drug-using populations, implementing outreach initiatives to overcome barriers to access. To achieve sustained reductions in the prevalence of HCV, it is crucial to enhance the affordability and availability of DAAs.

The results of this meta-analysis underscore the essential need for thorough, region-specific strategies to tackle the dual challenges posed by HBV and HCV in the drug-using population. Through the implementation of evidence-based strategies, the enhancement of access to prevention and treatment, and the tackling of structural inequities, public health stakeholders have the potential to achieve significant advancements in decreasing disease transmission and enhancing health outcomes for one of Asia’s most at-risk populations. This coordinated strategy shows potential for addressing the considerable public health issues related to HBV and HCV among drug-using communities throughout the area.

## 5. Strengths and Limitations

This study presents a thorough meta-analysis of HBV and HCV prevalence among drug users in Asia, integrating data from various settings and subpopulations. The study demonstrates a significant strength through its strict compliance with PRISMA guidelines, which guarantees methodological rigor and reproducibility. Incorporating both published and grey literature reduces the likelihood of publication bias and strengthens the dependability of aggregated estimates. Subgroup analyses yield detailed insights into geographic, temporal, and methodological variations, facilitating a nuanced understanding of prevalence patterns throughout the region.

Nonetheless, the study does have its limitations. The considerable heterogeneity observed across the included studies, especially in subgroup analyses, indicates notable variability in methodologies, populations, and settings. Retrospective studies frequently utilize selective sampling, which can lead to an inflation of prevalence estimates, whereas cross-sectional studies might not accurately reflect the true burden. The dependence on ELISA in numerous studies prompts questions regarding diagnostic specificity, especially for HBV, where molecular techniques such as PCR provide enhanced sensitivity. Finally, the lack of geographic diversity in the studies, particularly with a focus on a specific continent, limits the applicability of the findings to the wider global context.

## 6. Conclusions

This systematic review and meta-analysis highlight the significant impact of HBV and HCV on drug users in Asia. The findings highlight significant geographic differences in prevalence, illustrating the intricate relationship between healthcare access, harm reduction strategies, and socioeconomic factors. Although there have been reductions in HBV prevalence in certain areas thanks to vaccination initiatives, the persistently elevated rates of HCV underscore the critical necessity for improved prevention and treatment strategies. The results highlight the necessity of incorporating harm reduction services, including needle exchange programs and opioid substitution therapy, into wider public health strategies. Increasing the availability of HBV vaccination and guaranteeing the affordability of direct-acting antivirals for HCV are essential measures in alleviating the disease burden.

## Figures and Tables

**Table 1:** Characteristic table of the included studies on the dual burden of Hepatitis B and C among drug users in Asia.

Author’s Name	Year of Publication	Country	Total HBV Sampled	HBV Positive	Total HCV Sampled	HCV Positive	Study Types	Method of Detection
Verachai et al. [31]	2002	Thailand	NR	NR	303	124	Cross sectional	Elisa/PCR
Akthar et al. [22]	2015	Malaysia	664	86	NR	NR	Retrospective	ELISA
Zhang et al. [8]	2015	Vietnam	NR	NR	1434	1273	Cross sectional	ELISA
Ruslin et al. [32]	2001	Malaysia	130	68	NR	NR	Retrospective	ELISA
Duogh et al. [9]	2018	Vietnam	NR	NR	603	404	Retrospective	ELISA
Ha Thi Tu [33]	2009	Vietnam	NR	NR	455	350	Retrospective	ELISA
Vu minh et al. [10]	2009	Vietnam	309	250	309	229	Retrospective	ELISA
Linh et al. [34]	2019	Vietnam	NR	NR	1886	242	Cross sectional	ELISA
Nicholas et al. [35]	2023	Vietnam	NR	NR	1444	875	Cross sectional	PCR
Barnaby et al. [36]	2022	Vietnam	7740	1321	5461	3157	Retrospective	ELISA
Ishizak et al. [37]	2017	Vietnam	760	81	NR	NR	Retrospective	ELISA
Ishizak et al. (a) [37]	2017	Vietnam	302	34	NR	NR	Retrospective	ELISA
Ishizak et al. (b) [37]	2017	Vietnam	389	43	NR	NR	Retrospective	ELISA
Desjarlais et al. [38]	2016	Vietnam	603	404	NR	NR	Retrospective	ELISA
Prasetyo et al. [39]	2013	Indonesia	375	128	375	12	Retrospective	ELISA
Dunford [40]	2012	Vietnam	NR	NR	1000	556	Retrospective	ELISA
Dunford et al. [41]	2012	Vietnam	1000	174	NR	NR	Retrospective	ELISA
Telan et al. [42]	2011	Philippines	NR	NR	62	62	Retrospective	ELISA
Mcfall et al. [43]	2017	India	NR	NR	6457	1901	Cross sectional	ELISA
Gupta et al. [44]	2017	India	NR	NR	194	89	Cross sectional	PCR
Solomon et al. [45]	2016	India	NR	NR	1042	293	Cross sectional	PCR
Hsieh et al. [46]	2016	Taiwan	566	87	NR	NR	Retrospective	Elisa/PCR
Lie et al. [47]	2014	China	NR	NR	370	154	Retrospective	ELISA
Amitis et al. [48]	2014	Iran	100	9	100	56	Cross sectional	ELISA
Abbasali et al. [49]	2014	Iran	539	65	39	71	Cross sectional	Elisa/PCR
Hsieh et al. [50]	2014	Taiwan	562	86	562	513	Cross sectional	Elisa/PCR
Chalana et al. [51]	2013	India	118	6	118	78	Cross sectional	ELISA
Basu et al. [52]	2012	India	NR	NR	103	47	Cross sectional	ELISA
Min et al. [53]	2013	Korea	318	21	318	154	Retrospective	ELISA
Alipour et al. [54]	2013	Iran	NR	NR	268	102	Cross sectional	ELISA
Yen et al. [55]	2012	Taiwan	NR	NR	1447	1318	Retrospective	ELISA
Sofian [56]	2012	Iran	153	46	153	91	Retrospective	ELISA
Davoodian et al. [57]	2009	Iran	249	12	249	196	Cross sectional	ELISA
Solomon et al. [58]	2008	India	912	101	912	566	Cross sectional	ELISA
Mohesen et al. [59]	2015	Iran	NR	NR	7568	3406	Cross sectional	ELISA
Rahman et al. [60]	2018	Bangladesh	NR	NR	90	34	Retrospective	ELISA
Sharhani et al. [61]	2017	Iran	NR	NR	606	332	Cross sectional	ELISA
Kermode et al. [62]	2016	India	NR	NR	821	307	Cross sectional	ELISA
Ramenzani et al. [48]	2014	iran	100	6	100	6	Cross sectional	ELISA
Goswami et al. [63]	2014	India	839	51	839	56	Retrospective	ELISA
Goswami et al. (a) [63]	2014	India	860	92	860	601	Retrospective	ELISA
Goswami et al. (b) [63]	2014	India	821	46	821	121	Retrospective	ELISA
Goswami et al. (c) [63]	2014	India	829	67	829	125	Retrospective	ELISA
Ghosh et al. [64]	2012	India	58	2	NR	NR	Retrospective	ELISA
Mahanta et al. [65]	2009	India	397	15	398	190	Retrospective	ELISA
Chu et al. [66]	2009	Taiwan	192	32	192	172	Retrospective	ELISA
Jindal et al. [67]	2008	India	157	28	157	53	Retrospective	ELISA
Liu et al. [68]	1997	China	NR	NR	85	78	Retrospective	ELISA
Zuo et al. [69]	2000	China	NR	NR	60	57	Retrospective	ELISA
Ma et al. [70]	2004	China	NR	NR	102	70	Retrospective	ELISA
Wang et al. [71]	2006	China	NR	NR	106	34	Retrospective	ELISA
Li et al. [72]	1994	China	NR	NR	197	187	Retrospective	ELISA
Gu et al. [73]	1995	China	NR	NR	407	247	Retrospective	ELISA
Kong et al. [74]	1995	China	NR	NR	144	99	Retrospective	ELISA
Zhou et al. [75]	1995	China	NR	NR	58	41	Retrospective	ELISA
Chen et al. [76]	2000	China	NR	NR	37	27	Retrospective	ELISA
Yang et al. [77]	2000	China	NR	NR	182	172	Retrospective	ELISA
Zhang et al. [78]	2000	China	NR	NR	125	96	Retrospective	ELISA
Jiang et al. [79]	2003	China	NR	NR	118	94	Retrospective	ELISA
Li et al. [80]	2004	China	NR	NR	381	261	Retrospective	ELISA
Liu et al. [81]	1997	China	NR	NR	60	16	Retrospective	ELISA
Yang et al. [82]	2000	China	NR	NR	107	54	Retrospective	ELISA
Zhuang et al. [83]	2000	China	NR	NR	285	14	Retrospective	ELISA
He et al. [84]	2006	China	NR	NR	90	57	Retrospective	ELISA
Fang et al. [85]	2001	China	NR	NR	284	115	Retrospective	ELISA
Mou et al. [86]	2008	China	NR	NR	498	133	Retrospective	ELISA
Yang et al. [87]	2001	China	NR	NR	177	62	Retrospective	ELISA
Ruan et al. [88]	2004	China	NR	NR	379	269	Retrospective	ELISA
Zhang et al. [89]	2005	China	NR	NR	123	95	Retrospective	ELISA
Xie et al. [90]	2006	China	NR	NR	370	276	Retrospective	ELISA
Tang et al. [91]	1994	China	NR	NR	53	25	Retrospective	ELISA
Shi et al. [92]	1994	China	NR	NR	121	79	Retrospective	ELISA
Wan et al. [93]	1997	China	NR	NR	119	27	Retrospective	ELISA
Yan et al. [94]	1997	China	NR	NR	543	398	Retrospective	ELISA
Mai et al. [95]	1999	China	NR	NR	142	112	Retrospective	ELISA
Wei et al. [96]	1999	China	NR	NR	86	58	Retrospective	ELISA
Yang et al. [97]	1999	China	NR	NR	419	320	Retrospective	ELISA
Hu et al. [98]	2000	China	NR	NR	120	89	Retrospective	ELISA
Gu et al. [99]	2002	China	NR	NR	205	177	Retrospective	ELISA
Huang et al. [100]	2003	China	NR	NR	2640	1409	Retrospective	ELISA
Chen et al. [101]	2004	China	NR	NR	716	289	Retrospective	ELISA
Li et al. [102]	2004	China	NR	NR	235	232	Retrospective	ELISA
Wang et al. [103]	2002	China	NR	NR	508	294	Retrospective	ELISA
Tang et al. [104]	1996	China	NR	NR	91	66	Retrospective	ELISA
Bi et al. [105]	1998	China	NR	NR	77	69	Retrospective	ELISA
Lin et al. [106]	1999	China	NR	NR	58	39	Retrospective	ELISA
Wu et al. [107]	1999	China	NR	NR	113	104	Retrospective	ELISA
Liang et al. [108]	2000	China	NR	NR	208	186	Retrospective	ELISA
Garten et al. [109]	2004	China	NR	NR	485	402	Retrospective	ELISA
Wang et al. [110]	2000	China	NR	NR	174	63	Retrospective	ELISA
Yang et al. [111]	2000	China	NR	NR	97	56	Retrospective	ELISA
Tang et al. [112]	2000	China	NR	NR	480	444	Retrospective	ELISA
Deng et al. [113]	2003	China	NR	NR	62	48	Retrospective	ELISA
Zhao et al. [114]	1999	China	NR	NR	102	34	Retrospective	ELISA
Peng et al. [115]	2006	China	NR	NR	252	222	Retrospective	ELISA
He et al. [116]	2004	China	NR	NR	87	26	Retrospective	ELISA
Hu et al. [117]	2003	China	NR	NR	276	96	Retrospective	ELISA
Zhaou et al. [118]	2005	China	NR	NR	101	47	Retrospective	ELISA
Zhaou et al. (a) [119]	2006	China	NR	NR	141	73	Retrospective	ELISA
Yin et al. [120]	1999	China	NR	NR	68	45	Retrospective	ELISA
Gao et al. [121]	2001	China	NR	NR	68	48	Retrospective	ELISA
Ji et al. [122]	2001	China	NR	NR	40	15	Retrospective	ELISA
Zhu et al. [123]	1999	China	NR	NR	64	14	Retrospective	ELISA
Chen et al. [124]	2003	China	NR	NR	265	79	Retrospective	ELISA
Zhou et al. [125]	2004	China	NR	NR	306	130	Retrospective	ELISA
Li et al. [126]	2005	China	NR	NR	114	71	Retrospective	ELISA
Wang et al. [127]	2001	China	NR	NR	32	5	Retrospective	ELISA
Zhang et al. [128]	2002	China	NR	NR	51	14	Retrospective	ELISA
Wang et al. [129]	2002	China	NR	NR	307	149	Retrospective	ELISA
Guo et al. [130]	2003	China	NR	NR	525	351	Retrospective	ELISA
Zhang et al. [131]	2006	China	NR	NR	232	122	Retrospective	ELISA
Jiang et al. [132]	2002	China	NR	NR	71	50	Retrospective	ELISA

Keynote: NR—Not reported.

**Table 2:** Subgroup meta-analysis of the prevalence of HBV among drug users in ASIA.

Parameter	No of Studies	Prevalence (%)	Confidence Interval (%)	Q	I^2^	Heterogeneity DF	*p*
Country							
Malaysia	2	28.7	5.4–74.0	90.295	98.89	1	<0.001
Vietnam	7	26.6	13.1–46.4	1070.995	99.44	6	<0.001
Indonesia	1	26.6	33.0–43.3	–	–	–	–
Taiwan	3	15.5	13.7–17.6	0.222	0	2	0.895
Iran	5	10.5	5.2–20.2	54.561	92.67	4	<0.001
Korea	1	6.6	4.3–9.9	–	–	–	–
India	9	7.7	5.8–10.2	58.719	86.38	8	<0.001
Study design							
Retrospective	21	16.7	11.5–23.6	1613.737	98.76	20	<0.001
Cross-sectional	7	9.5	7.1–12.5	26.312	77.2	6	<0.001
Method of detection							
ELISA	25	14.3	14.3–100.0	1695.51	98.58	24	<0.001
ELISA/PCR	3	14.3	12.3–16.6	3.188	37.26	2	0.203
Year of publication							
2011–2015	15	10.9	7.8–15.1	329.84	95.76	14	<0.001
2001–2005	1	52.3	43.7–60.7	–	–	–	–
2006–2010	6	16	4.3–44.5	504.76	99.01	5	<0.001
2021–2024	1	17.1	16.2–17.9	–	–	–	–
2016–2020	5	19.4	6.2–46.9	582.795	99.31	4	<0.001

**Table 3:** Subgroup analysis of the pooled prevalence of HBV among drug users in relation to country, study designs, and year of publication.

Parameter	No of Studies	Prevalence (%)	Confidence Interval (%)	Q	I^2^	Heterogeneity DF	*p*
Country							
Vietnam	7	63.5	44.3–79.2	1606.126	99.63	6	<0.001
Thailand	1	40.9	35.5–46.6	-	-	-	-
Indonesia	1	3.2	1.8–5.5	-	-	-	-
Philippines	1	99.2	88.5–100.0	-	-	-	-
India	13	35.8	26.1–46.9	1321.086	99.09	12	<0.001
Iran	8	49.3	39.8–58.8	172.606	95.94	7	<0.001
China	66	62.9	58.0–67.7	2065.8	96.85	65	<0.001
Iran	8	49.3	39.8–58.8	172.606	95.94	7	<0.001
Taiwan	3	91	89.7–92.1	0.536	0	2	0.765
Korea	1	48.4	43.0–53.9	-	-	-	-
Bangladesh	1	37.8	28.4–48.2	-	-	-	-
Study design							
Retrospective	83	60.4	55.5–65.1	4343.258	98.11	82	<0.001
Cross-sectional	19	50	40.6–59.4	2751.01	99.35	18	<0.001
Method of detection							
ELISA/PCR	3	67.4	24.8–92.9	208.093	99.04	2	<0.001
ELISA	96	58.6	54.0–63.0	7462.608	98.73	95	<0.001
PRC	3	44.4	23.1–68.0	245.267	99.18	2	<0.001
Year of publication							
2001–2010	41	57.1	49.7–64.5	4230.883	99.05	40	<0.001
2011–2020	28	47.6	35.3–59.9	19,174.396	99.86	27	<0.001
>2020	2	59	56.3–61.7	3.695	72.94	1	0.055
<2001	31	65.6	54.1–77.2	4239.471	99.29	30	<0.001

## Data Availability

The raw data supporting the conclusions of this article will be made available by the authors on request.

## Supplementary Materials

The following supporting information can be downloaded at: https://www.mdpi.com/article/10.3390/pathogens14040360/s1, File S1: Search strategy; File S2: JBI quality assessment of the included studies.
