# Supplementary material for: The Dual Burden of Hepatitis B and C Among Drug Users in Asia: The First Systematic Review and Meta-Analysis
Source: Pathogens. 2025 Apr 7;14(4):360. doi: 10.3390/pathogens14040360 (PMC12030361; doi:10.3390/pathogens14040360)
Supplement: Supplementary file 1 [file pathogens-14-00360-s001.zip › pathogens-3554250-supplementary/M1-SEARCH STRATEGY FOR HBV_HCV.pdf]

SCOPUS

TITLE-ABS-KEY("hepatitis B" OR HBV OR "hepatitis C" OR HCV)
AND TITLE-ABS-KEY("dual burden" OR co-infection OR "combined infection")
AND TITLE-ABS-KEY("drug users" OR "people who inject drugs" OR PWID OR "substance use disorder")
AND TITLE-ABS-KEY(Asia OR Afghanistan OR Bangladesh OR Bhutan OR Brunei OR Cambodia OR China OR India OR Indonesia OR Japan OR Kazakhstan OR Korea OR Laos OR Malaysia OR Maldives OR Mongolia OR Myanmar OR Nepal OR Pakistan OR Philippines OR Singapore OR Sri Lanka OR Tajikistan OR Thailand OR Timor-Leste OR Turkmenistan OR Uzbekistan OR Vietnam).

SCIENCE DIRECT

("hepatitis B" OR HBV OR "hepatitis C" OR HCV)
AND ("dual burden" OR coinfection OR "combined infection")
AND ("drug users" OR "people who inject drugs" OR PWID OR "substance use disorder")
AND (Asia OR Afghanistan OR Bangladesh OR Bhutan OR Brunei OR Cambodia OR China OR India OR Indonesia OR Japan OR Kazakhstan OR Korea OR Laos OR Malaysia OR Maldives OR Mongolia OR Myanmar OR Nepal OR Pakistan OR Philippines OR Singapore OR Sri Lanka OR Tajikistan OR Thailand OR Timor-Leste OR Turkmenistan OR Uzbekistan OR Vietnam).

PUBMED

("Hepatitis B"[MeSH Terms] OR "HBV"[All Fields] OR "Hepatitis C"[MeSH Terms] OR "HCV"[All Fields]) AND ("Coinfection"[MeSH Terms] OR ("coinfect"[All Fields] OR "coinfected"[All Fields] OR "coinfecting"[All Fields] OR "Coinfection"[MeSH Terms] OR "Coinfection"[All Fields] OR "coinfections"[All Fields] OR "coinfects"[All Fields]) OR "dual burden"[All Fields] OR "combined infection"[All Fields]) AND ("substance abuse, intravenous"[MeSH Terms] OR "drug users"[All Fields] OR "people who inject drugs"[All Fields] OR "PWID"[All Fields] OR "substance use disorder"[All Fields]) AND ("Asia"[MeSH Terms] OR ("afghanistan"[MeSH Terms] OR "afghanistan"[All Fields] OR "afghanistan s"[All Fields]) OR ("bangladesh"[MeSH Terms] OR "bangladesh"[All Fields] OR "bangladesh s"[All Fields]) OR

("bhutan"[MeSH Terms] OR "bhutan"[All Fields] OR "bhutan s"[All Fields]) OR ("brunei"[MeSH Terms] OR "brunei"[All Fields]) OR ("cambodia"[MeSH Terms] OR "cambodia"[All Fields] OR "cambodia s"[All Fields]) OR ("china"[MeSH Terms] OR "china"[All Fields] OR "china s"[All Fields] OR "chinas"[All Fields]) OR ("india"[MeSH Terms] OR "india"[All Fields] OR "india s"[All Fields] OR "indias"[All Fields]) OR ("indonesia"[MeSH Terms] OR "indonesia"[All Fields] OR "indonesia s"[All Fields] OR "indonesias"[All Fields]) OR ("japan"[MeSH Terms] OR "japan"[All Fields] OR "japan s"[All Fields] OR "japans"[All Fields]) OR ("kazakhstan"[MeSH Terms] OR "kazakhstan"[All Fields] OR "kazakhstan s"[All Fields]) OR ("korea"[MeSH Terms] OR "korea"[All Fields] OR "korea s"[All Fields] OR "koreas"[All Fields]) OR ("laos"[MeSH Terms] OR "laos"[All Fields]) OR ("malaysia"[MeSH Terms] OR "malaysia"[All Fields] OR "malaysia s"[All Fields]) OR ("maldive"[All Fields] OR "maldives"[MeSH Terms] OR "maldives"[All Fields]) OR ("mongolia"[MeSH Terms] OR "mongolia"[All Fields] OR "mongolia s"[All Fields]) OR ("myanmar"[MeSH Terms] OR "myanmar"[All Fields] OR "myanmar s"[All Fields] OR "myanmars"[All Fields]) OR ("nepal"[MeSH Terms] OR "nepal"[All Fields] OR "nepal s"[All Fields]) OR ("pakistan"[MeSH Terms] OR "pakistan"[All Fields] OR "pakistan s"[All Fields]) OR ("philippine"[All Fields] OR "philippines"[MeSH Terms] OR "philippines"[All Fields]) OR ("singapore"[MeSH Terms] OR "singapore"[All Fields] OR "singapore s"[All Fields]) OR ("sri lanka"[MeSH Terms] OR ("sri"[All Fields] AND "lanka"[All Fields]) OR "sri lanka"[All Fields]) OR ("tajikistan"[MeSH Terms] OR "tajikistan"[All Fields]) OR ("thailand"[MeSH Terms] OR "thailand"[All Fields] OR "thailand s"[All Fields]) OR ("timor leste"[MeSH Terms] OR "timor leste"[All Fields] OR ("timor"[All Fields] AND "leste"[All Fields]) OR "timor leste"[All Fields]) OR ("turkmenistan"[MeSH Terms] OR "turkmenistan"[All Fields]) OR ("uzbekistan"[MeSH Terms] OR "uzbekistan"[All Fields]) OR ("vietnam"[MeSH Terms] OR "vietnam"[All Fields] OR "vietnam s"[All Fields]))

GOOGLE SCHOLAR

"hepatitis B" OR HBV OR "hepatitis C" OR HCV

AND "dual burden" OR coinfection OR "combined infection"

AND "drug users" OR "people who inject drugs" OR PWID OR "substance use disorder"

AND (Asia OR Afghanistan OR Bangladesh OR Bhutan OR Brunei OR Cambodia OR China OR India OR Indonesia OR Japan OR Kazakhstan OR Korea OR Laos OR Malaysia OR Maldives OR

Mongolia OR Myanmar OR Nepal OR Pakistan OR Philippines OR Singapore OR Sri Lanka OR Tajikistan OR Thailand OR Timor-Leste OR Turkmenistan OR Uzbekistan OR Vietnam).

WEB OF SCIENCE

TS=("hepatitis B" OR HBV OR "hepatitis C" OR HCV)
AND TS=("dual burden" OR coinfection OR "combined infection")
AND TS=("drug users" OR "people who inject drugs" OR PWID OR "substance use disorder")
AND TS=(Asia OR Afghanistan OR Bangladesh OR Bhutan OR Brunei OR Cambodia OR China OR India OR Indonesia OR Japan OR Kazakhstan OR Korea OR Laos OR Malaysia OR Maldives OR Mongolia OR Myanmar OR Nepal OR Pakistan OR Philippines OR Singapore OR Sri Lanka OR Tajikistan OR Thailand OR Timor-Leste OR Turkmenistan OR Uzbekistan OR Vietnam).

GREY DATABASES (WHO)

"Hepatitis B and C dual burden among drug users in Asia"
"Coinfection HBV HCV in drug users in Asia"
"Substance use and viral hepatitis in Asia"
