# Supplementary material for: The Dual Burden of Hepatitis B and C Among Drug Users in Asia: The First Systematic Review and Meta-Analysis
Source: Pathogens. 2025 Apr 7;14(4):360. doi: 10.3390/pathogens14040360 (PMC12030361; doi:10.3390/pathogens14040360)
Supplement: Supplementary file 1 [file pathogens-14-00360-s001.zip › pathogens-3554250-supplementary/M2-HBV_HCV QUALITY OF INCLUDED STUDIES BY JBI CRITICAL APPRAISAL CHECKLIST FOR STUDIES REPORTING PREVALENCE DATA.pdf]

QUALITY OF INCLUDED STUDIES BY JBI CRITICAL APPRAISAL CHECKLIST FOR STUDIES REPORTING PREVALENCE DATA

S/N	Name of authors [reference] and year of publication		JBI checklist*									Total
1	Verachai et al [31]	2002	1	2	3	4	5	6	7	8	9	
2	Akthar et al[32]	2015	Yes	No	Yes	Yes	Yes	Yes	Yes	Yes	Yes	16
3	Zhang et al [33]	2015	Yes	No	Yes	Yes	Yes	Yes	Yes	Yes	Yes	16
4	Ruslin et al [34]	2001	Yes	No	Yes	Yes	Yes	Yes	Yes	Yes	Yes	16
5	Duogh et al [35]	2018	Yes	No	Yes	Yes	Yes	Yes	Yes	Yes	Yes	16
6	Ha Thi Tu [36]	2009	Yes	No	Yes	Yes	Yes	Yes	Yes	Yes	Yes	16
7	Vu minh et al [37]	2009	Yes	No	Yes	Yes	Yes	Yes	Yes	Yes	Yes	16
8	Linh et al [38]	2019	Yes	No	Yes	Yes	Yes	Yes	Yes	Yes	Yes	16
9	Nicholas et al [39]	2023	Yes	No	Yes	Yes	Yes	Yes	Yes	Yes	Yes	16
10	Barnaby et al [40]	2022	Yes	No	Yes	Yes	Yes	Yes	Yes	Yes	Yes	16
11	Ishizak et al [41]	2017	Yes	No	Yes	Yes	Yes	Yes	Yes	Yes	Yes	16
12	Ishizak et al (a) [41]	2017	Yes	No	Yes	Yes	Yes	Yes	Yes	Yes	Yes	16
13	Ishizak et al (b) [41]	2017	Yes	No	Yes	Yes	Yes	Yes	Yes	Yes	Yes	16
14	Desjarlais et al [42]	2016	Yes	No	Yes	Yes	Yes	Yes	Yes	Yes	Yes	16
15	Prasetyo et al [43]	2013	Yes	No	Yes	Yes	Yes	Yes	Yes	Yes	Yes	16
16	Dunford [44]	2012	Yes	No	Yes	Yes	Yes	Yes	Yes	Yes	Yes	16
17	Dunford et al [45]	2012	Yes	No	Yes	Yes	Yes	Yes	Yes	Yes	Yes	16
18	Telan et al [46]	2011	Yes	No	Yes	Yes	Yes	Yes	Yes	Yes	Yes	16
19	Mcfall et al [47]	2017	Yes	No	Yes	Yes	Yes	Yes	Yes	Yes	Yes	16
20	Gupta et al [48]	2017	Yes	No	Yes	Yes	Yes	Yes	Yes	Yes	Yes	16
21	Solomon et al [49]	2016	Yes	No	Yes	Yes	Yes	Yes	Yes	Yes	Yes	16
22	Hsieh et al [50]	2016	Yes	No	Yes	Yes	Yes	Yes	Yes	Yes	Yes	16
23	Lie et al [51]	2014	Yes	No	Yes	Yes	Yes	Yes	Yes	Yes	Yes	16
24	Amitis et al [52]	2014	Yes	No	Yes	Yes	Yes	Yes	Yes	Yes	Yes	16
25	Abbasali et al [53]	2014	Yes	No	Yes	Yes	Yes	Yes	Yes	Yes	Yes	16
26	Hsieh et al [54]	2014	Yes	No	Yes	Yes	Yes	Yes	Yes	Yes	Yes	16
27	Chalana et al [55]	2013	Yes	Yes	Yes	Yes	Yes	Yes	Yes	Yes	Yes	18
28	Basu et al [56]	2012	Yes	Yes	Yes	Yes	Yes	Yes	Yes	Yes	Yes	18
29	Min et al [57]	2013	Yes	No	Yes	Yes	Yes	Yes	Yes	Yes	Yes	16
30	Alipour et al [58]	2013	Yes	No	Yes	Yes	Yes	Yes	Yes	Yes	Yes	16
31	Yen et al [59]	2012	Yes	No	Yes	Yes	Yes	Yes	Yes	Yes	Yes	16
32	Sofian [60]	2012	Yes	No	Yes	Yes	Yes	Yes	Yes	Yes	Yes	16
33	Davoodian et al [61]	2009	Yes	No	Yes	Yes	Yes	Yes	Yes	Yes	Yes	16
34	Solomon et al [62]	2008	Yes	No	Yes	Yes	Yes	Yes	Yes	Yes	Yes	16
35	Mohesen et al [63]	2015	Yes	No	Yes	Yes	Yes	Yes	Yes	Yes	Yes	16
36	Rahman et al [64]	2018	Yes	No	Yes	Yes	Yes	Yes	Yes	Yes	Yes	16
37	Sharhani et al [65]	2017	Yes	Yes	Yes	Yes	Yes	Yes	Yes	Yes	Yes	18
38	Kermode et al [66]	2016	Yes	No	Yes	Yes	Yes	Yes	Yes	Yes	Yes	16
39	Ramenzani et al [67]	2014	Yes	Yes	Yes	Yes	Yes	Yes	Yes	Yes	Yes	18
40	Goswami et al [68]	2014	Yes	No	Yes	Yes	Yes	Yes	Yes	Yes	Yes	16
41	Goswami et al (a) [68]	2014	Yes	Yes	Yes	Yes	Yes	Yes	Yes	Yes	Yes	18
42	Goswami et al (b)[68]	2014	Yes	No	Yes	Yes	Yes	Yes	Yes	Yes	Yes	16
43	Goswami et al (c.)[68]	2014	Yes	No	Yes	Yes	Yes	Yes	Yes	Yes	Yes	16
44	Ghosh et al [69]	2012	Yes	No	Yes	Yes	Yes	Yes	Yes	Yes	Yes	16
45	Mahanta et al [70]	2009	Yes	Yes	Yes	Yes	Yes	Yes	Yes	Yes	Yes	18
46	Chu et al [71]	2009	Yes	No	Yes	Yes	Yes	Yes	Yes	Yes	Yes	16
47	Jindal et al [72]	2008	Yes	Yes	Yes	Yes	Yes	Yes	Yes	Yes	Yes	18
48	Liu et al [73]	1997	Yes	No	Yes	Yes	Yes	Yes	Yes	Yes	Yes	16

49	Zuo et al [74]	2000	Yes	Yes	Yes	Yes	Yes	Yes	Yes	Yes	Yes	18
50	Ma et al [75]	2004	Yes	No	Yes	Yes	Yes	Yes	Yes	Yes	Yes	16
51	Wang et al [76]	2006	Yes	No	Yes	Yes	Yes	Yes	Yes	Yes	Yes	16
52	Li et al [77]	1994	Yes	No	Yes	Yes	Yes	Yes	Yes	Yes	Yes	16
53	Gu et al [78]	1995	Yes	No	Yes	Yes	Yes	Yes	Yes	Yes	Yes	16
54	Kong et al [79]	1995	Yes	Yes	Yes	Yes	Yes	Yes	Yes	Yes	Yes	18
55	Zhou et al [80]	1995	Yes	Yes	Yes	Yes	Yes	Yes	Yes	Yes	Yes	18
56	Chen et al [81]	2000	Yes	No	Yes	Yes	Yes	Yes	Yes	Yes	Yes	16
57	Yang et al [82]	2000	Yes	No	Yes	Yes	Yes	Yes	Yes	Yes	Yes	16
58	Zhang et al [83]	2000	Yes	No	Yes	Yes	Yes	Yes	Yes	Yes	Yes	16
59	Jiang et al[84]	2003	Yes	No	Yes	Yes	Yes	Yes	Yes	Yes	Yes	16
60	Li et al [85]	2004	Yes	No	Yes	Yes	Yes	Yes	Yes	Yes	Yes	16
61	Liu et al [86]	1997	Yes	No	Yes	Yes	Yes	Yes	Yes	Yes	Yes	16
62	Yang et al [87]	2000	Yes	No	Yes	Yes	Yes	Yes	Yes	Yes	Yes	16
63	Zhuang et al [88]	2000	Yes	No	Yes	Yes	Yes	Yes	Yes	Yes	Yes	16
64	He et al [89]	2006	Yes	No	Yes	Yes	Yes	Yes	Yes	Yes	Yes	16
65	Fang et al [90]	2001	Yes	No	Yes	Yes	Yes	Yes	Yes	Yes	Yes	16
66	Mou et al [91]	2008	Yes	No	Yes	Yes	Yes	Yes	Yes	Yes	Yes	16
67	Yang et al [92]	2001	Yes	No	Yes	Yes	Yes	Yes	Yes	Yes	Yes	16
68	Ruan et al [93]	2004	Yes	No	Yes	Yes	Yes	Yes	Yes	Yes	Yes	16
69	Zhang et al [94]	2005	Yes	No	Yes	Yes	Yes	Yes	Yes	Yes	Yes	16
70	Xie et al [95]	2006	Yes	No	Yes	Yes	Yes	Yes	Yes	Yes	Yes	16
71	Tang et al[96]	1994	Yes	No	Yes	Yes	Yes	Yes	Yes	Yes	Yes	16
72	Shi et al [97]	1994	Yes	No	Yes	Yes	Yes	Yes	Yes	Yes	Yes	16
73	Wan et al [98]	1997	Yes	No	Yes	Yes	Yes	Yes	Yes	Yes	Yes	16
74	Yan et al [99]	1997	Yes	No	Yes	Yes	Yes	Yes	Yes	Yes	Yes	16
75	Mai et al [100]	1999	Yes	No	Yes	Yes	Yes	Yes	Yes	Yes	Yes	16
76	Wei et al [101]	1999	Yes	No	Yes	Yes	Yes	Yes	Yes	Yes	Yes	16
77	Yang et al [102]	1999	Yes	No	Yes	Yes	Yes	Yes	Yes	Yes	Yes	16
78	Hu et al [103]	2000	Yes	No	Yes	Yes	Yes	Yes	Yes	Yes	Yes	16
79	Gu et al [104]	2002	Yes	No	Yes	Yes	Yes	Yes	Yes	Yes	Yes	16
80	Huang et al [105]	2003	Yes	No	Yes	Yes	Yes	Yes	Yes	Yes	Yes	16
81	Chen et al [106]	2004	Yes	No	Yes	Yes	Yes	Yes	Yes	Yes	Yes	16
82	Li et al [107]	2004	Yes	No	Yes	Yes	Yes	Yes	Yes	Yes	Yes	16
83	Wang et al [108]	2002	Yes	No	Yes	Yes	Yes	Yes	Yes	Yes	Yes	16
84	Tang et al [109]	1996	Yes	No	Yes	Yes	Yes	Yes	Yes	Yes	Yes	16
85	Bi et al [110]	1998	Yes	No	Yes	Yes	Yes	Yes	Yes	Yes	Yes	16
86	Lin et al [111]	1999	Yes	No	Yes	Yes	Yes	Yes	Yes	Yes	Yes	16
87	Wu et al [112]	1999	Yes	No	Yes	Yes	Yes	Yes	Yes	Yes	Yes	16
88	Liang et al [113]	2000	Yes	No	Yes	Yes	Yes	Yes	Yes	Yes	Yes	16
89	Garten et al [114]	2004	Yes	No	Yes	Yes	Yes	Yes	Yes	Yes	Yes	16
90	Wang et al [115]	2000	Yes	No	Yes	Yes	Yes	Yes	Yes	Yes	Yes	16
91	Yang et al [116]	2000	Yes	No	Yes	Yes	Yes	Yes	Yes	Yes	Yes	16
92	Tang et al [117]	2000	Yes	No	Yes	Yes	Yes	Yes	Yes	Yes	Yes	16
93	Deng et al [118]	2003	Yes	No	Yes	Yes	Yes	Yes	Yes	Yes	Yes	16
94	Zhao et al [119]	1999	Yes	No	Yes	Yes	Yes	Yes	Yes	Yes	Yes	16
95	Peng et al [120]	2006	Yes	No	Yes	Yes	Yes	Yes	Yes	Yes	Yes	16
96	He et al [121]	2004	Yes	No	Yes	Yes	Yes	Yes	Yes	Yes	Yes	16
97	Hu et al [122]	2003	Yes	No	Yes	Yes	Yes	Yes	Yes	Yes	Yes	16
98	Zhaou et al [123]	2005	Yes	No	Yes	Yes	Yes	Yes	Yes	Yes	Yes	16
99	Zhaou et al (a) [124]	2006	Yes	No	Yes	Yes	Yes	Yes	Yes	Yes	Yes	16
100	Yin et al [125]	1999	Yes	No	Yes	Yes	Yes	Yes	Yes	Yes	Yes	16
101	Gao et al [126]	2001	Yes	No	Yes	Yes	Yes	Yes	Yes	Yes	Yes	16

102	Ji et al [127]	2001	Yes	No	Yes	Yes	Yes	Yes	Yes	Yes	Yes	16
103	Zhu et al [128]	1999	Yes	No	Yes	Yes	Yes	Yes	Yes	Yes	Yes	16
104	Chen et al [129]	2003	Yes	No	Yes	Yes	Yes	Yes	Yes	Yes	Yes	16
105	Zhou et al [130]	2004	Yes	No	Yes	Yes	Yes	Yes	Yes	Yes	Yes	16
106	Li et al [131]	2005	Yes	No	Yes	Yes	Yes	Yes	Yes	Yes	Yes	16
107	Wang et al [132]	2001	Yes	No	Yes	Yes	Yes	Yes	Yes	Yes	Yes	16
108	Zhang et al [133]	2002	Yes	No	Yes	Yes	Yes	Yes	Yes	Yes	Yes	16
109	Wang et al [134]	2002	Yes	No	Yes	Yes	Yes	Yes	Yes	Yes	Yes	16
110	Guo et al [135]	2003	Yes	No	Yes	Yes	Yes	Yes	Yes	Yes	Yes	16
111	Zhang et al [136]	2006	Yes	No	Yes	Yes	Yes	Yes	Yes	Yes	Yes	16
112	Jiang et al [137]	2002	Yes	No	Yes	Yes	Yes	Yes	Yes	Yes	Yes	16

JBI CHECKLIST* 1. Appropriate sampling frame to address target population, **2.** Appropriate sampling way of study participants, **3.** Adequate sample size, **4.** Detail description of study participants and settings, **5.** Data analysis with sufficient coverage of identified sample, **6.** Use of valid methods to identify the condition, **7.** Standard, reliable way of measurement of condition for all participants, **8.** Availability of appropriate statistical analysis, **9.** Adequate response rate and management of low response rate.

Scores are coded as Yes=2 and No=0.
